# Linear and Volumetric Mandibular Asymmetries in Adult Patients With Different Skeletal Classes and Vertical Patterns: A Cone-Beam Computed Tomography Study

**DOI:** 10.1038/s41598-018-30270-7

**Published:** 2018-08-17

**Authors:** Luz Victoria Mendoza, Carlos Bellot-Arcís, José María Montiel-Company, Verónica García-Sanz, José Manuel Almerich-Silla, Vanessa Paredes-Gallardo

**Affiliations:** 1 Department of Orthodontics, Faculty of Medicine and Dentistry, University of Valencia, Valencia, Spain; 2 Department of Preventive Dentistry, Faculty of Medicine and Dentistry, University of Valencia, Valencia, Spain

## Abstract

This study aimed to quantify the height of the mandibular condyle and ramus, condylar volume, and the asymmetry index in adult patients of different sex, skeletal class and vertical pattern using Cone-Beam Computed Tomography (CBCT), and to determine whether there were differences between these groups. The study used CBCT scans of 159 patients with a mean age of 32.32 ± 8.31 years. InVivoDental® software was used to perform both linear (condylar, ramal, and total height) and condylar volume measurements. Linear and volumetric asymmetries were calculated. There were not significant differences between right and left sides. The mean value obtained for condyle height was 7.27 mm, ramus height 42.3 mm, total height 49.6 mm and condyle volume 1907.1 mm^3^, with significant differences between men and women. Significantly higher values were found for condylar volume in hypodivergent patterns (p = 0.001) and for the asymmetry index of the condylar volume in Class II patients (p < 0.05). The prevalence of relevant asymmetry was high for condyle height and volume (73.1% y 75.6% respectively). Higher height and volume values were found among men, Class III, and hypodivergent patients. Linear and volumetric asymmetries were more prevalent among men, Class III and hyperdivergent patterns.

## Introduction

Mandibular asymmetry has been described as dimensional differences in size, form, and volume of the left and right side of the mandible^1^, which can be the cause of aesthetic and functional problems^2^.

Different techniques including clinical examination, photography, and radiography have been used to assess mandibular asymmetry^3,4^. Traditionally, the most commonly used have been two-dimensional (2D) radiographs, including posteroanterior (PA) cephalograms^5,6^, submentovertex^7^ and panoramic radiographs^1,8–11^.

In order to measure mandibular asymmetries quantitatively from panoramic radiographs, various techniques have been proposed, Habets’^12^ and Kjellberg’s^13^ methods being the most frequently used. The method proposed by Habets *et al*.^12^ is considered a reliable method for quantitatively assessing vertical mandibular asymmetry. According to these authors, asymmetry index values higher than 3% on panoramic images may be referred to as mandibular asymmetry^12^.

The associations between mandibular asymmetry and different factors such as TMDs^6,14^, posterior crossbite^8,10,15^, cleft lip and palate^16–18^ and different occlusion patterns^2,7,17–22^ have been evaluated in several studies using Habets’ method^12^.

Panoramic radiographs present some vertical magnification and other distortion problems derived from projection geometry, which can lead to inaccurate evaluations and limit their diagnostic usefulness^23^. It has been noted that the reproducibility of vertical measures and angles will be acceptable providing the head is positioned correctly when taking x-rays. Habets *et al*.^12^ only considered asymmetries relevant when the asymmetry index was higher than 3%, which was associated with a 6% difference between sides when measured on panoramic radiographs.

Cone-Beam Computed Tomography (CBCT) is an accurate and reliable method for assessing craniofacial structures^24,25^, providing a three-dimensional (3D) reconstruction of anatomical structures with high resolution and no magnification^26^. Lim *et al*. compared the mandibular asymmetry index using panoramic radiography and CBCT finding low reliability and validity for panoramic radiographs, thus recommending CBCT^27^. Only one published study has evaluated condylar and ramal vertical asymmetries using CBCT in patients with different vertical growth patterns but with normal, Class I sagittal skeletal patterns^28^. Meanwhile, volumetric measurements of the jaw and the mandibular condyle have been reported in CBCT images of Caucasian patients^29,30^ and in patients with juvenile arthritis^31^. Only one study has described volume according to the vertical and anteroposterior skeletal pattern (in a Japanese population) but without taking into account the symmetry or asymmetry that subjects presented^32^.

The aims of this study were firstly, to quantify the height of the mandibular ramus and condyle, condylar volume and the asymmetry index in adult patients of different sex, skeletal class and vertical pattern using Cone Beam Computed Tomography (CBCT), and secondly, to determine whether there were differences between these groups.

## Materials and Methods

The study protocol was approved by the Ethics Committee for Research Involving Human Subjects at the University of Valencia, Spain (H1465893129760). Rights were protected by the Institutional Review Board. All subjects gave their informed consent to take part in the study. Any data that might disclose the identity of the participants have been omitted. This study was designed following guidelines established in the Helsinki declaration and the STROBE statement^33^.

### Sample

CBCT scans of patients attending the orthodontic clinic at the University of Valencia (Spain) between January 2015 and March 2017 were obtained from the clinic’s archives. All CBCT scans were taken for diagnostic reasons relating to dental treatment, so the patients did not receive any additional radiation for the purpose of the present study. A total of 195 patient’s records were selected by VPG and LVM. After receiving information about the study, a total of 189 patients were willing to take part. Six patients did not wish to participate for personal reasons. The positive response rate was 96.9%. Informed consent was obtained from all participants.

All CBCT scans were taken using a Planmeca Promax 3D imaging device (Planmeca, Helsinki, Finland) and included images of the complete skull (field of view 20 × 19 cm) with a voxel size of 0.4 mm. The scans were taken with the patient’s head in its natural position and the lips and tongue in the resting position.

Inclusion criteria were:

1. Patients who were to undergo any dental treatment;
2. Patients with a CBCT as part of their general dental records taken before the patient underwent any treatment;
3. Caucasian patients;
4. Patients with all dentition present from first lower molar to first lower molar on the contralateral side;
5. Patients older than 25 years, no longer in mandibular growth.

Exclusion criteria were:

1. Patients with any craniofacial anomalies or syndromes;
2. Patients with antecedents of any kind of trauma to the mandible;
3. Patients with presence of any type of cross-bite and/or mandibular functional shifting caused by occlusal interferences;
4. Patients with presence of any type of temporomandibular disorder (TMD).

Power analysis showed that a sample size of 159 patients would provide an 80% probability of detecting a medium effect (f = 0.25) for differences in the asymmetry index between skeletal classes or vertical patterns using an ANOVA model at a confidence level of 95%.

## Methods

Lateral cephalogram radiographs were extracted from the CBCT images using Dolphin Imaging software in order to classify the patients according to the following parameters:

1. Anteroposterior skeletal class (I, II or III). Steiner ANB angle was used to classify patients by skeletal Class: Class I presenting values of 2 ± 2°; Class II presenting values >4°, and Class III presenting values < 0°^34^.
2. Vertical pattern: this refers to the vertical position of the mandible with respect to the cranial base: hypodivergent, normal, hyperdivergent. This was determined by Ricketts XY axis angle^35^ (Normal = 90 ± 3°; Hyperdivergent <87°; Hypodivergent >93°).

CBCT images were imported from the software InVivoDental® 5.1 (Anatomage®, San Jose, California, USA) and both linear and volumetric measurements were performed.

### Linear measurements (CH, RH and CH + RH)

**Figure 1:**
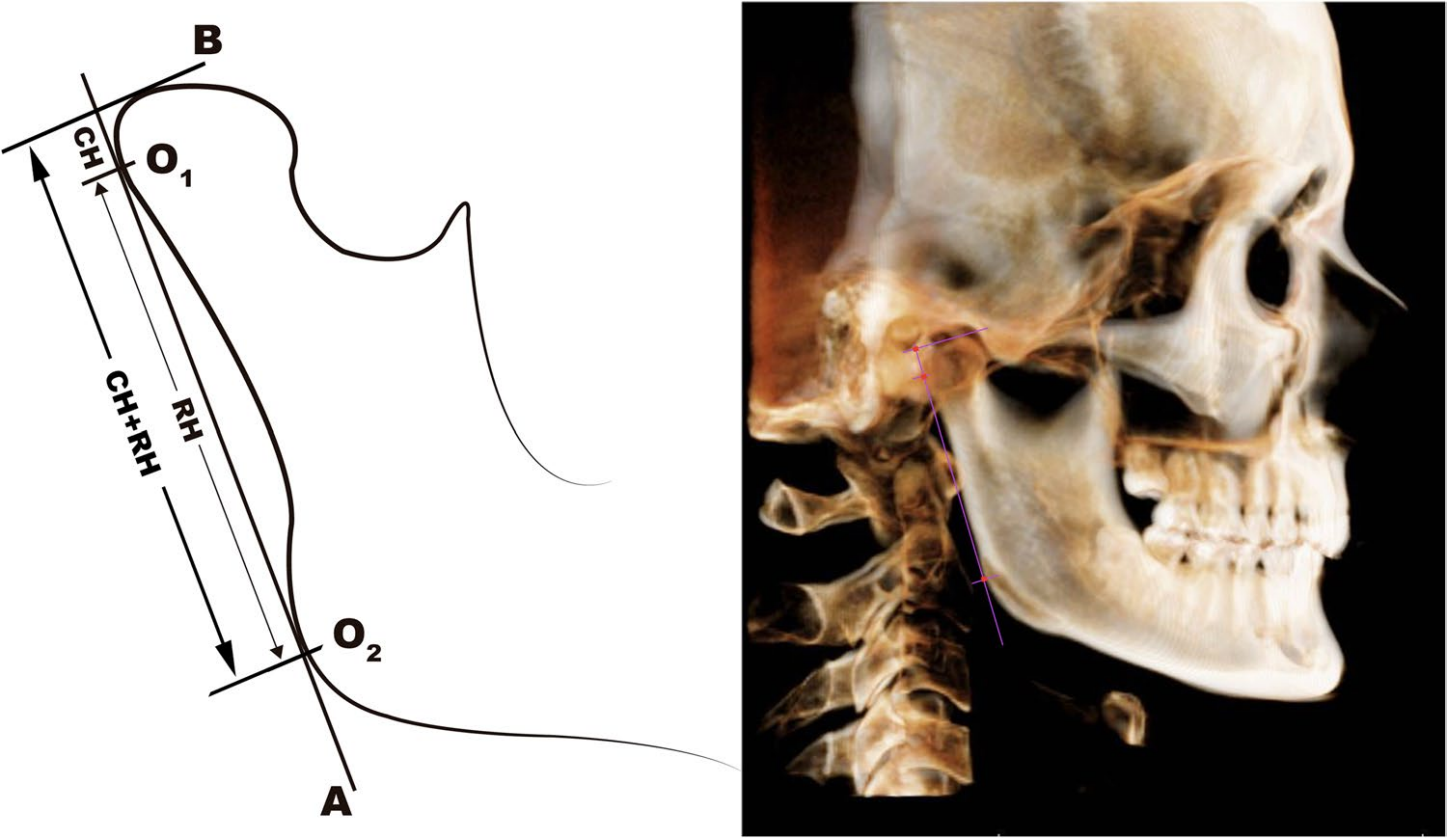
Linear measurements method. Performance of CH, RH and CH + RH measurements.

Condylar (CH), ramal (RH) and total (condylar plus ramal height; CH + RH) were measured on both sides of each mandible using Habets’ method^12^ as shown in Fig. 1. The most posterior points of the condyle and ramus were marked (O_1_ and O_2_) and a line was drawn through them (A-line). Another line, (B-line) was drawn from the most superior point of the condyle perpendicular to the A-line. Firstly, the distance between point O_1_ and the intersection point of A and B lines was measured, representing condylar height (CH). Secondly, the distance between O_1_ and O_2_ representing ramal height (RH). Lastly, the distance between point O_2_ and the intersection point of A and B lines was measured, representing total height (CH + RH). Vertical mandibular asymmetry indexes of the condyle, ramus, and condyle plus ramus were calculated using the formula developed by Habets *et al*.^12^: Asymmetry index (%): [(Right − Left)/(Right + Left)] × 100.

### Volumetric measurements (CVol)

Condylar volume (CVol), comprising the head and neck of the condyle, was measured as follows: on the sagittal view, the condylar area was delimited by identifying the lowest point of the sigmoid notch and a line was drawn through it, parallel to the Frankfort plane, which was constructed separately for each side by identifying right and left Porion and Orbitale points. The delimited condylar structure was isolated from the rest of the image using the software’s cropping tool. The threshold value was set based on the best visualization of the structure.

**Figure 2:**
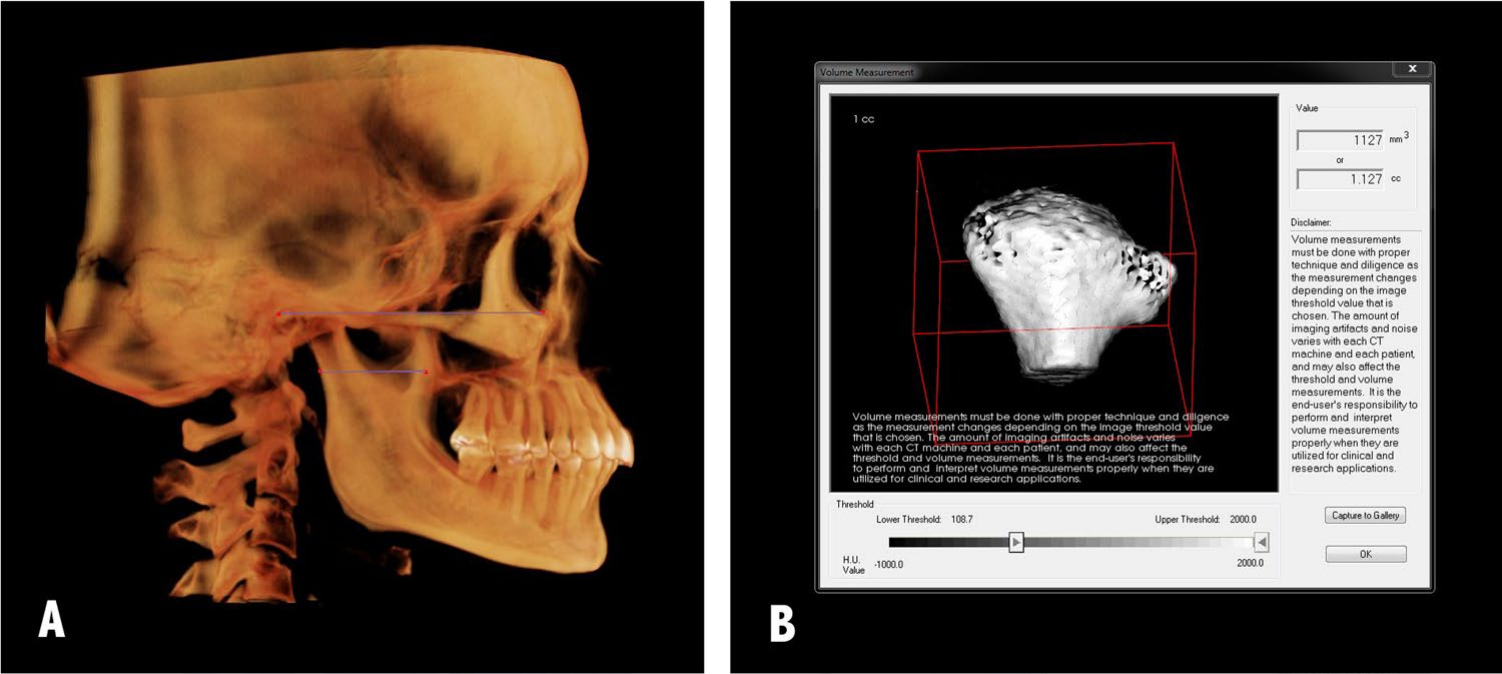
Condylar volume measurement method. (**A**) Delimitation of the condylar area. (**B**) Volume measurement.

The volume-measuring tool was used to determine the volume of the isolated structure in mm^3^ (Fig. 2). The same formula used to determine the vertical asymmetry of the mandible was applied to estimate condylar volume asymmetry: [(Right − Left)/(Right + Left)] × 100. Only asymmetries over 3% were considered to be relevant in accordance with the threshold value established by Habets *et al*.^12^.

### Statistical analysis

To calculate intra-observer reproducibility, the same observer (LM) repeated measurements of 100 CBCTs, with a 1-week period between the first and second measurements. Inter-observer reproducibility was evaluated by a second observer (VP), instructed and calibrated, who repeated the measurements of 100 CBCTs. Both observers redefined all the points before taking the second measurement, including the landmarks for sigmoid notch and Frankfort Plane. The two examiners chose their own threshold settings. Both linear and volumetric measurements were repeated. In this way, measurement error could be estimated, as well as intra- and inter-observer reproducibility. The Dahlberg formula (d) was used to estimate error, calculating the coefficient of variation (CV) as an indicator of relative error or measuring technique. The data obtained were entered on a spreadsheet, using Microsoft Excel 2011 (Microsoft Corp, Redmond, Wash) and transferred to the statistical software package SPSS v. 22.0 for analysis.

To analyze the agreement between the measurements of the right and left sides, the Passing Bablok regression line^36^ and Lin’s coefficient of agreement^37^ were used. The existence of differences in means was determined by the Paired t-test.

Independent t-test was used to test differences in means of linear and volumetric measurements, and asymmetry index between male and female patients. One-way analysis of variance (ANOVA) models were used to study independently differences involving skeletal class or vertical pattern. Tuckey test was used as post hoc test. Differences in the prevalence of relevant asymmetries (asymmetry index >3%) were estimated using the Chi-squared test.

To analyze the associations regarding asymmetry prevalence (asymmetry index with cut-off points of >1%, >3%, >6% and >10%) of linear and volumetric measurements with the independent variables (sex, skeletal class and vertical pattern), a multivariate analysis by logistic regression was conducted with the Forward Selection Method.

Level of significance α = 0.05 was set up for the analysis.

### Data availability

The datasets generated during and/or analyzed during the current study are available from the corresponding author on reasonable request.

## Results

After applying the inclusion and exclusion criteria, the final sample consisted of 159 patients (74 males and 85 females) with a mean age of 32.32 ± 8.31 years, ranging from 24.34 to 41.21 years.

The final sample comprised the following:

- 61 patients with skeletal Class I: 29 with normal vertical pattern (10 men and 19 women); 19 with hypodivergent pattern (8 men and 11 women); and 13 with hyperdivergent pattern (3 men and 11 women). Their mean age was 33.65 ± 12.39 years.
- 54 patients with skeletal Class II: 22 with normal vertical pattern (12 men and 10 women); 16 with hypodivergent pattern (9 men and 7 women); and 16 with hyperdivergent pattern (7 men and 9 women). Their mean age was 33.13 ± 12.42 years.
- 44 patients with skeletal Class III: 14 with normal vertical pattern (8 men and 6 women); 10 with hypodivergent pattern (6 men and 4 women); and 20 with hyperdivergent pattern (11 men and 9 women). Their mean age was 29.63 ± 11.91years.

Sex distribution was found to be homogeneous, unlike vertical pattern distribution. The numbers of CBCT scans pertaining to hyperdivergent and hypodivergent patients were similar, and slightly higher than the number of patients with normal pattern.

Reproducibility results showed an intra-observer coefficient of variation (CV) of between 0.70% and 1.13%; the inter-observer CV ranged between 1.21% and 1.49. Intra and inter-observer error measurement method for *d* of Dahlberg fell below 0.16 mm.

**Figure 3:**
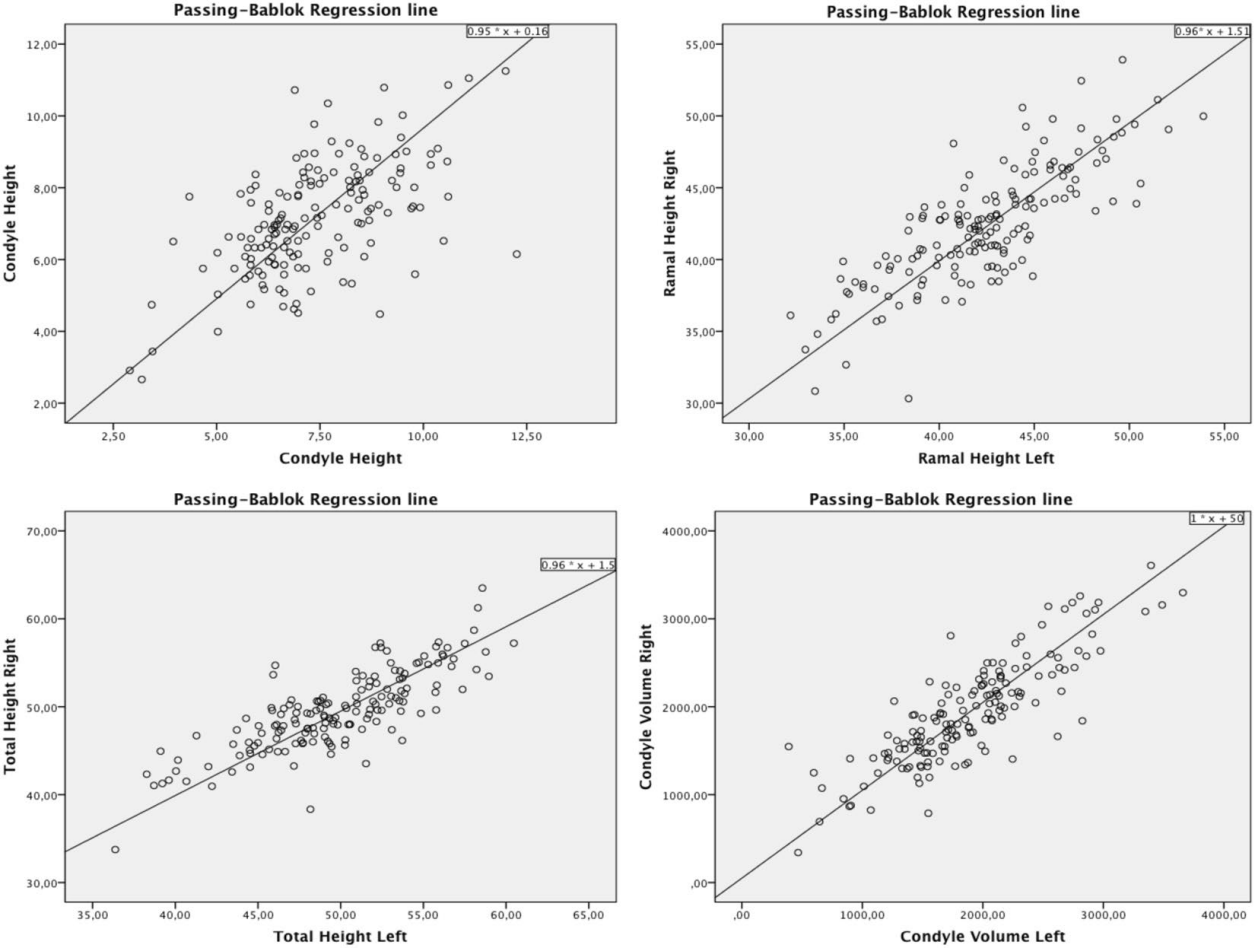
Passing Bablok regression lines for linear and volumetric measurements.

No differences were found by the paired t-test in the means of condyle height, ramus height, total height and condyle volume between the right and the left side. Lin’s coefficient showed high agreement in all measurements except for condyle height, which was moderate (Table 1). The Passing Bablok regression line did not determine the existence of constant or proportional differences between sides (Fig. 3).

**Table 1:** Linear (condylar height, ramus height, total height) and volumetric measurements (condylar volume) according to the side (right and left).

		Right Side	Left Side	Difference of means	Paired T Test p valor	Lin’s Coefficient Agreement
Linear Measurements	Condyle Height (CH) mm (CI 95%)	7.15 (6.91_7.39)	7.37 (7.11_7.63)	−0.21 (−0.44_0.02)	p = 0.070	0.582 (0.470_0.672)
	Ramus Height (RH) mm (CI 95%)	42.3 (41.7_42.9)	42.4 (41.7_43.0)	−0.02 (−0.43_0.40)	p = 0.939	0.799 (0.735_0.849)
	Total Height (CH + RH) mm (CI 95%)	49.5 (48.8_50.2)	49.7 (48.9_50.5)	−0.25 (−0.71_0.21)	p = 0.281	0.802 (0.739_0.851)
Volumetric Measurements	Condyle Volume (o) mm^3^ (CI 95%)	1932.7 (1841.1_2024.2)	1881.6 (1789.0_1974.1)	51,1 (−0.62_102.8)	p = 0.053	0.840 (0.787_0.881)

Linear (condylar height, ramus height, total height) and volumetric measurements (condylar volume) according to gender, skeletal class and vertical pattern are shown in Table 2. Values were significantly higher for men than for women in all measurements. Total height mean value was significantly lower for skeletal class I when compared to skeletal class III (p = 0.021). Condyle volume was significantly higher for hypodivergent than for hyperdivergent patterns (p = 0.001).

**Table 2:** Linear (condylar height, ramus height, total height) and volumetric measurements (condylar volume) according to gender, skeletal class and vertical pattern.

		Linear Measurements			Volumetric Measurements
		Condyle Height (CH) mm (CI 95%)	Ramus Height (RH) mm (CI 95%)	Total Height (CH + RH) mm (CI 95%)	Condyle Volume (CV) mm^3^ (CI 95%)
Total n = 159		7.27 (7.04–7.48)	42.3 (41.7–43.0)	49.6 (48.9–50.3)	1907.1 (1818.8–1995.5)
Gender	Male n = 74	7.51 (7.21–7.82)	44.2 (43.4–45.2)	51.7 (50.8–52.7)	2135.2 (2007.6–2262.7)
	Female n = 85	7.04 (6.71–7.36)	40.6 (40.0–41.4)	47.7 (46.9–48.5)	1708.6 (1608.0–1816.3)
	Student t test p value	p = 0.037	p = 0.000	p = 0.000	p = 0.000
Skeletal Class	Class I n = 61	7.14 (6.81–7.47)	41.5 (40.7–42.4)	48.6 (47.8–49.6)	1874.3 (1730.7–2018.0)
	Class II n = 54	7.12 (6.68–7.53)	42.5 (42.4–43.6)	49.5 (48.2–50.8)	1879.3 (1707.6–2051.1)
	Class III n = 44	7.60 (7.17–8.01)	43.4 (42.1–44.6)	51.0 (49.6–52.4)	1986.7 (1839.9–2133.4)
	ANOVA p value Tukey post-hoc	p = 0.184	p = 0.059	p = 0.028* p = 0.021 I vs. III	p = 0.548
Vertical Pattern	Normal n = 65	7.32 (7.05–7.61)	42.5 (41.6–43.4)	49.9 (48.9–50.8)	1901.8 (1784.6–2118.9)
	Hypodivergent n = 45	7.50 (7.05–7.94)	42.5 (41.3–43.7)	50.0 (48.7–51.3)	2130.9 (1963.6–2298.2)
	Hyperdivergent n = 49	6.95 (6.47–7.44)	42.0 (40.7–43.2)	48.9 (47.4–50.4)	1708.6 (1532.2–1885.1)
	ANOVA p value Tukey post-hoc	p = 0.165	p = 0.711	p = 0.415	p = 0.001* p = 0.001 hyper vs hypo

Regarding asymmetry index for both linear and volumetric measurements according to gender, skeletal class and vertical pattern (Table 3), statistically significant differences were only found for condylar volume when comparing skeletal classes, being higher for Class II (p < 0.05).

**Table 3:** Asymmetry index of the linear and volumetric measurements according to gender, skeletal class and vertical pattern.

Asymmetry Index		Linear Measurements			Volumetric Measurements
		Condyle height (CH) mean (CI 95%)	Ramus height (RH) mean (CI 95%)	Total height (CH + RH) mean (CI 95%)	Condyle Volume (CVol) mean (CI 95%)
Total n = 159		−1.39 (−2.97_0.19)	−0.01 (−0.50_0.49)	−0.22 (−0.69_0.24)	1.57 (−0.04_3.18)
Gender	Male n = 74	−2.38 (−4.81_0.04)	0.11 (−0.66_0.88)	−0.27 (−0.96_0.42)	1.13 (−0.85_3.11)
	Female n = 85	−0.53 (−2,62_1,57)	−0.12 (−0.76_0.53)	−0.18 (−0.83_0.46)	1.95 (−0.56_4.47)
	Student t test p value	p = 0.407	p = 0.738	p = 0.801	p = 0.298
Skeletal Class	Class I n = 61	−2.09 (−4.58_0.39)	0.15 (−0.61_0.91)	−0.28 (−0.97_0.42)	0.33 (−1.83_2.50)
	Class II n = 54	0.60 (−2.18_3.39)	−0.37 (−1.24_0.51)	−0.18 (−1.04_0.67)	4.89 (1.67–8.11)
	Class III n = 44	−2.87 (−6.00_0.26)	0.22 (−0.80_1.23)	−0.21 (−1.19_0.77)	−0.78 (−3.79_2.23)
	ANOVA p value Tukey post-hoc	p = 0.188	p = 0.587	p = 0.985	p = 0.012* p < 0.05 II vs I and III
Vertical Pattern	Normal n = 65	−2.86 (−5.42_−0.30)	−0.18 (−0.96_0.60)	−0.59 (−1.31_0.13)	−0.01 (−2.23_2.21)
	Hypodivergent n = 45	−0.34 (−2.88_2.20)	−0.25 (−1.19_0.68)	−0.29 (−1.19_0.61)	1.97 (−0.45_4.39)
	Hyperdivergent n = 49	−0,41 (−3.58_2.77)	0.44 (−0.48_1.36)	0.32 (−0.55_1.19)	3.31 (−0.51_7.14)
	ANOVA p value	p = 0.315	p = 0.485	p = 0.271	p = 0.225

The 75.6% of the patients showed condylar volume asymmetry, and the 73.1% presented asymmetries in condylar height. The prevalence in ramus height asymmetry was 32.5%, this prevalence being 26.3% for total height.

No associations were found between the prevalence of asymmetries (linear or volumetric) and sex, skeletal class or vertical pattern (Table 4).

**Table 4:** Percentage of individuals with asymmetry (asymmetry index >3%) according to gender, skeletal class and vertical pattern.

Asymmetry %		Linear Measurements			Volumetric Measurements
		Condyle height (CH) % (CI 95%)	Ramus height (RH) % (CI 95%)	Total height (CH + RH) % (CI 95%)	Condyle Volume (CVol) % (CI 95%)
Total n = 159		73.1 (65.7–79.4)	32.5 (25.7–40.1)	26.3 (20.0–33.6)	75.6 (68.4–81.6)
Gender	Male n = 74	76.6 (66.0–84.7)	35.1 (25.3–46.2)	29.9 (20.8–40.8)	76.6 (66.0–84.7)
	Female n = 85	69.9 (59.3–78.7)	30.1 (21.3–40.7)	22.9 (15.2–33.0)	74.7 (64.4–82.8)
	Chi^2^ p value	p = 0.336	p = 0.504	p = 0.316	p = 0.776
Skeletal Class	Class I n = 61	68.9 (56.4–79.1)	31.1 (20.9–43.6)	21.3 (12.9–33.1)	73.8 (61.6–83.2)
	Class II n = 54	71.7 (58.4–82.0)	34.0 (22.7–47.4)	28.3 (17.9–41.6)	69.8 (56.5–80.5)
	Class III n = 44	80.4 (66.8–89.3)	32.6 (20.9–47.0)	30.4 (19.1–44.8)	84.8 (71.7–92.4)
	Chi^2^ p value	p = 0.392	p = 0.949	p = 0.522	p = 0.204
Vertical Pattern	Normal n = 65	78.8 (67.5–86.9)	28.8 (19.3–40.6)	24.2 (15.5–35.8)	71.2 (59.4–80.7)
	Hypodivergent n = 45	68.8 (54.3–80.5)	28.9 (17.7–43.4)	26.7 (15.9–41.0)	77.8 (63.7–87.5)
	Hyperdivergent n = 49	69.4 (55.5–80.5)	40.8 (28.2–54.7)	28.6 (17.8–42.4)	79.6 (66.4–88.5)
	Chi^2^ p value	p = 0.399	p = 0.328	p = 0.870	p = 0.541

Table 5 shows the results of the multivariate analysis by logistic regression, where the prevalence of individuals with asymmetry at different cut-off points (>1%, >3%, >6%, >10%) was analysed. Only the association between condylar volume asymmetry >10% and hyperdivergent pattern (odds ratio = 2.65; IC-95 = 1.20–5.84) and between condylar height asymmetry >10% and skeletal class III (odds ratio = 2.88; IC-95% = 1.36–6.08) were found to be significant.

**Table 5:** Percentage of individuals with asymmetry (asymmetry index with cut-off points >1%, >3%, >6%, >10%).

Asymmetry %	Linear Measurements			Volumetric Measurements
	Condyle height (CH) % (CI 95%)	Ramus height (RH) % (CI 95%)	Total height (CH + RH) % (CI 95%)	Condyle Volume (CVol) % (CI 95%)
Cut-off 1%	91.2 (85.8–94.7)	71.7 (64.2–78.1)	71.7 (64.2–78.1)	94.3 (89.6–97.0)
Cut-off 3%	73.0 (65.6–79.3)	32.7 (25.9–40.3)	27.0 (20.7–34.4)	76.1 (68.9–82.1)
Cut-off 6%	49.1 (41.4–56.8)	3.8 (1.7–8.0)	4.4 (2.1–8.8)	45.9 (38.4–53.7)
Cut-off 10%	27.0*^a^ (20.7–34.4)	0.6 (0.1–3.5)	0.6 (0.1–3.5)	20.8*^b^ (15.2–27.7)

*^a^Associated with logistic regression by Forward Selection (Wald) to Class III (OR = 2.882; p = 0.005).

*^b^Associated with logistic regression by Forward Selection (Wald) to hyperdivergent pattern (OR = 2.652; p = 0.015).

## Discussion

This study provides information about CH, RH, CH + RH, CVol, linear and volumetric asymmetries in adult patients of different sex, skeletal class and vertical pattern, in order to determine whether there are differences between these groups. Establishing the differences (if any) in mandible dimensions and asymmetries between patients with different skeletal characteristics could help our understanding of the etiology of mandibular asymmetries hence establish an accurate treatment plan.

The sample was homogeneous regarding genders and skeletal classes. Only when making combinations between the variables, this homogeneity decreased. However, this was not considered a problem since the aim of the study was not to make associations between sex, skeletal class and vertical pattern variables. The only combination that exhibited heterogeneity was class I with hyperdivergent pattern (3 men and 11 women), which was not considered a limitation since the logistical regression conducted in the present study did not find significant associations between sex and the prevalence of asymmetries (Table 5).

Unlike the original study^12^ and other subsequent research^38^, the present study took measurements from CBCTs rather than panoramic radiographs in order to avoid magnification and distortion problems^17^, and to provide high resolution^26^. All the CBCTs used in this study derived from patients’ dental records, and had been taken for other purposes (implants, supernumerary teeth, third molar surgery, etc.), and not specifically for the study.

The ANB angle^34^ was used to determine skeletal class as in previous studies^1,28,32,38^. Ricketts’ XY axis angle^35^ was used to classify patients according to vertical pattern, unlike other studies that have used the mandibular plane^28,32^; this particular factor may constitute a difference between studies. The reason why the authors used Ricketts’ XY axis angle was to avoid mismatched planes resulting from mandibular plane differences between right and left sides, which could affect the measurements. Since Ricketts uses Gnation point, the authors considered this angle to be more reliable. Other studies on craniofacial structures classify the patients and make associations based on phenotypic clusters thus allowing clinical interpretation^39,40^. However, studies on mandibular shape and asymmetries classify patients according to vertical pattern and skeletal class like in our study, which allow more reliable comparisons between studies^1,28,32,38^

In the present study, the patients selected did not present any type of crossbite, as it has been shown that the mandibular structure presents significant asymmetries within crossbite groups^8,10,15,17^.

The mandible undergoes maximum growth months after the maximum pubertal outbreak of growth and continues for two years after the cessation of maxillary growth^41^. The mean age of all the patients in the present study was 32.32 years, implying that the mandible was no longer in growth, so that growth would not affect measurements.

The patients selected for this study presented full permanent dentition from the first lower molar to the first lower molar on the contralateral side, as there is a proven relation between absence of the first lower molar and vertical mandibular asymmetry^1^.

Patients with any kind of temporomandibular disorder (TMD) were also excluded from the study since TMD is associated with mandibular asymmetry^6,14^.

The study sample only included Caucasian patients in order to avoid complications arising from ethnicity. This could explain why the results may not coincide with studies that investigated Turkish^28,42^, Chilean^38,43^, Chinese^44^, and Japanese^32^ populations.

Patients with any craniofacial anomalies or syndromes such as cleft lip and palate were also excluded from this study, as these too are associated with mandibular asymmetry^16–18^.

In this type of study, the accuracy and reliability of the imaging software used could be an important factor affecting the results. The present study used InVivoDental® software, unlike other investigations that have used Visualization Toolkit package, MIRIT, Matlab FastRBF Toolbox, Ortho Pro 2.0 software^45^, Simplant OMS^46^, Mimics^TM^^29,30,44^, Simplant Pro version 13.0^28^, Analyze^32^.

Intra- and inter-observer error was low and so reproducibility was high, the coefficient of variation value (CV) and the Dahlberg d-value being very low.

Even though differences between right and left sides were found, they were not statistically significant, a finding that concurs with the results of other studies^1,8,28,47^. It should be noted that, unlike the present study, some studies have used panoramic radiographs, and included patients with crossbite and mandibular shifting, factors that could affect the results^1,28^. Although differences were not significant, a high percentage of patients presented relevant linear asymmetries, condyle height asymmetry being particularly prevalent. The present study also found a high prevalence of volumetric asymmetry, although differences did not reach statistical significance. These results differ from others^44,45^. The differences could be due to the different inclusion criteria applied in the latter studies, in which these authors^44^ used a sample of patients diagnosed with mandibular asymmetry, and analyzed patients in mixed dentition^45^.

Linear measurements were found to be higher among men than women, with greater difference in the ramus. These differences were statistically significant, a finding that partly agrees with Saglam study^42^ which found significant differences in the total asymmetry index between men and women. Contrarily to our results, other studies did not find significant differences^1,2,10,18,21,47^.

In the present study, Class III patients showed higher linear measurement values, while other researchers have found the mandibular vertical dimension to be higher among Class II patients^38,48^. Regarding asymmetries in the different skeletal classes, Class III patients showed greater condylar asymmetries, while greater ramus asymmetries were found among Class II patients, although these differences were not statistically significant. Kasimoglu *et al*.^47^ also analyzed these differences, and like the present results did not find statistically significant differences. However, Saglam^42^ did find significant differences in total vertical asymmetry between skeletal classes. As for condylar volume, the present study found significant differences in asymmetry index between skeletal classes, which was higher for Class II patients, this finding being in contrast with Nakawaki *et al*. study which did not find significant differences among skeletal classes^32^. This disagreement may be owed to the ethnic differences between the samples, since their study uses a sample of Japanese patients unlike our study.

Hyperdivergent patients showed lower linear values, although without statistically significant differences. These results agree with Celik *et al*., who conducted a similar study to the present one, although the patient sample was smaller^28^. Hyperdivergent patients showed higher rates of asymmetry compared with the other groups, again without statistically significant differences; these results are in agreement with Celik *et al*.^28^. Significant differences in condylar volume were found between different vertical patterns. In this regard, our results agree with Nakawaki *et al*.^32^, who did find significant differences between hyperdivergent and hypodivergent patients, whereby the hypodivergent group presented higher volumes.

By applying a multivariate analysis with logistic regression, the present study aimed to analyse the associations between different grades of asymmetry and the other studied parameters. Other authors have used different statistical tools such as geometric morphometric analysis to study asymmetries^49^

The present study suffered certain limitations. Firstly, the method described by Habets et al^12^. does not delimit the condylar head accurately, so condyle height measurement is an estimation of its length based on the location of cephalometric points. Secondly, although measurements taken from CBCTs are accurate, volume measurements may be subject to variations resulting from varying bone densities, which can affect the segmentation process^50^. Some 3D computational shape analysis methods are nowadays available to accurately assess anatomical aspects of the craniofacial complex^49,51^. These methods also allow to make precise superimpositions of the structures after performing semi-automated segmentation, thus asymmetries can be quantified^52,53^. De Dumast *et al*. have recently described the Shape Variation Analyzer, which consists in an interesting tool for this matter^54^. Further research would be interesting to analyze whether there are differences in means of results between the method used in the present study and the modern 3D segmentation analyses.

Despite selecting patients without any craniofacial anomalies, previous trauma, crossbite, mandibular shifting, or any kind of TMD, the present study found a high prevalence of relevant asymmetry within the sample, which points to the importance of assessing asymmetries during diagnosis in order to ensure accurate and comprehensive treatment planning.

## Conclusions

Greater height and volume values were found among men, Class III, and hypodivergent patients. Linear and volumetric asymmetries were more prevalent among men, Class III and hyperdivergent patterns. Significant associations were found between condylar volume asymmetries >10% and hyperdivergent pattern, and between condylar height asymmetries >10% and skeletal class III.
